# Morning cortisol as an alternative to Short Synecthan test for the diagnosis of primary adrenal insufficiency

**DOI:** 10.12669/pjms.35.5.1208

**Published:** 2019

**Authors:** Imran Ulhaq, Tauseef Ahmad, Adeel Khoja, Najmul Islam

**Affiliations:** 1 Dr. Imran Ulhaq, FCPS. Consultant Endocrinologist Aga Khan University Hospital, Karachi, Pakistan; 2 Dr. Tauseef Ahmad, FCPS. Assistant Professor of Medicine/Endocrinology, Dr. Ziauddin University Hospital, Karachi, Pakistan; 3 Dr. Adeel Khoja, M.Sc, (Epidemiology and Biostatistics), Aga Khan University Hospital, Karachi, Pakistan; 4 Dr. Najmul Islam, FRCP. Aga Khan University Hospital, Karachi, Pakistan

**Keywords:** Morning cortisol, Primary adrenal insufficiency, Short synacthan test, Single center

## Abstract

**Objective:**

To determine 7-9 am serum cortisol less than 5mcg/dl is an independent reliable confirmatory test for the diagnosis of primary adrenal insufficiency (PAI).

**Methods:**

A total of 164 patients who visited the outpatient or inpatient department of Aga Khan University Hospital from June 2011 to June 2017 were included for the study. All those patients whose levels came out less than 5mcg/dl were recruited for the study and they all underwent SST. Other demographic and laboratory data were also recorded.

**Results:**

The sensitivity of morning cortisol for diagnosis of PAI is 100% if levels are <1mcg/dl and decreases to 71.88% if levels are up to 5mcg/dl.

**Conclusion:**

Morning cortisol is sensitive enough as an alternative to SST if levels are <1mcg/dl (100%). However, if the levels are increased from > 1mcg/dl to < 5 mcg/dl, the sensitivity decreases gradually from 98% to 71%.

## INTRODUCTION

Cortisol hormone contribute significantly to maintaining homeostasis particularly through their role in the regulation of the body’s adaptive response to stress, in the maintenance of body water, electrolyte balance, and in the control of blood pressure; The Adrenal cortex synthesizes the steroid hormones called glucocorticoids (cortisol), mineralocorticoids (aldosterone) and androgens in response to hypothalamic-pituitary-adrenal hormone stimulation via adrenocorticotrophin hormone (ACTH). The ACTH release cortisol in a pulsatile manner with approximately 7–15 episodes daily. The stimulation of cortisol release occurs within 15 minutes of the surge in ACTH.^1^

In addition to being pulsatile, the release of cortisol also follows a circadian rhythm. Release of cortisol is greatest during early morning waking hours, with levels declining as the afternoon progresses. This has a direct impact on how cortisol values are interpreted according to the timing of blood sample collection. The release of ACTH from the anterior pituitary is regulated by the hypothalamus via corticotropin-releasing hormone (CRH). Cortisol inhibits the secretion of ACTH in a negative feedback regulation mechanism. This closely regulated circuit is referred to as the hypothalamic-pituitary-adrenal (HPA) axis.

Primary adrenal insufficiency (PAI) or Addison’s disease is defined by the inability of the adrenal cortex to produce sufficient amount of glucocorticoids and/or mineralocorticoids^2^ is a potentially life-threatening condition due to the central role of these hormones in energy, salt, and fluid homeostasis.

The symptoms of PAI are quite nonspecific and include generalized body weakness, fatigue, body aches, nausea, vomiting, abdominal pain, depression, and anxiety. As a result, the diagnosis is frequently delayed, resulting in a clinical presentation of acute life-threatening adrenal crisis.^3^

PAI is a rare disease with a reported prevalence of about 100 to 140 cases per million and an incidence of 4:1,000,000 per year in Western societies; autoimmunity is the most common cause of PAI^4^-^7^, followed by infectious diseases such as tuberculosis, adrenalectomy, neoplasia, and various genetic causes.

According to guidelines, primary adrenal insufficiency (PAI) is screened by 8am serum cortisol level and confirmed by ACTH stimulation test^2^, the sensitivity of this test for the diagnosis of PAI is 92%. As there is a lot of financial constraints in our country and there is often non-availability of cosyntropin. Our study aim is to establish whether 7-9am cortisol less than five is an independent reliable confirmatory test for the diagnosis of primary adrenal insufficiency.

## METHODS

This retrospective study was conducted at Department of Medicine in collaboration with section of Chemical Pathology, Department of Pathology & Laboratory Medicine, Aga Khan University Hospital (AKUH), and Karachi, Pakistan from June 2011 to June 2017. Study was commenced after taking approval from institution’s ethical review committee (ERC). All patients with cortisol level of less than 5mcg/dl that underwent ACTH stimulation test at AKUH laboratory were included. Patient admitted in intensive care unit (ICU) were excluded. Cut off used was post synacthen stimulation was more than 20mcg/dl.

### Data analysis

Descriptive analysis consisted of reporting mean with standard deviation (for symmetrically distributed variables) or median with interquartile range (for asymmetrically distributed variables) for quantitative (continuous) variables such as age, cortisol levels at baseline, 0 minutes and 60 minutes post synacthen injections. Frequency with percentages was reported for all categorical variable such as gender. Chi-square test was used to compare proportions across categories (0 mcg/dl to 5 mcg/dl) for adrenal insufficiency. Two sample paired t-test was used to compare pre and post short synacthen tests. The variable of baseline cortisol level was divided into six categories (0 to <1 mcg/dl, l to <2 mcg/dl, 2 to <3 mcg/dl, 3 to <4 mcg/dl, 4 to <5 mcg/dl, 5 mcg/dl) while the variable of post short synacthen test was categorized into <20 mcg/dl and > 20 mcg/dl. Chi-square test was used to compare these two above mentioned variables. Moreover, proportions and percentages were also reported for each respective category. A p-value <0.05 was considered as statistically significant. All analyses were conducted using the Statistical package for Social Sciences SPSS (version 19.0, copyright © SPSS; 1989-02).

## RESULTS

A total of 164 subjects were included in the study. The mean age of the subjects was 48±15.24 years. 61% (n=100) of the subjects were female while 39% (n=64) being male.

Mean baseline cortisol level before the synacthen test was 2.77±0.12mcg/dl. Mean cortisol level 60 minutes after post-synacthen test was 12.85±0.58mcg/dl. Mean difference between baseline cortisol and 60 minutes post-synacthen test was 10.075mcg/dl which was highly significant (*p*-value <0.001) (Table-I). 140 (85%) study participants had post-short synacthen cortisol level < 20mcg/dl while 24 (15%) of them had post-synacthen cortisol level > 20 mcg/dl (Table–II).

**Table I:** Pre & post short synacthen tests (two sample paired t-test).

S. No.	Variables	Mean±S.D.	Mean Difference	p-value
1.	Baseline cortisol level	2.77±0.12	10.075	< 0.001
2.	Post synacthen cortisol level	12.85±0.58		

**Table II:** Post-short synacthen cortisol level on stimulation with Tetracosynacthen (ACTH) after 60 mins as per standard guidelines^2^.

S. No.	Post-short synacthen cortisol levels	No. (%)
1.	< 20 mcg/dl	140 (85%)
2.	≥20 mcg/dl	24 (15%)

Out of the total study participants (11.5%) having baseline cortisol levels of 0 mcg/dl to <1mcg/dl with respect to post-synacthen stimulation test after sixty minutes were 100% (n=19) which showed inadequate response. Moreover, 27.4% (n=45) of the total study participants had baseline cortisol level of 1 mcg/dl to < 2mcg/dl, while 97.7% (n=44) had post-synacthen stimulation test as inadequate response after sixty minutes and 2.2% (n=1) showed normal response after sixty minutes of the same category. Out of the total study participants, 14.6 % (n=24) had baseline cortisol levels of 2 mcg/dl to < 3mcg/dl, among them 83.3% (n=20) subjects showed inadequate response while 16.6% (n=4) showed normal response after sixty minutes. In the category of baseline cortisol levels of 3 mcg/dl to < 4mcg/dl, 16.4 % (n=27) of total study subjects were falling in the same range, among them 81.4% (n=22) had post-synacthen stimulation test as inadequate response after sixty minutes while 18.5% (n=5) showed normal response after sixty minutes. Moreover, 19.5% (n=32) of the total study subjects had baseline cortisol levels of 4mcg/dl to < 5mcg/dl, among them, 71.8% (n=23) had post-synacthen stimulation as inadequate response, while 28% (n=9) showed normal response after sixty minutes. Lastly, 10.6% (n=17) of the total study subjects had a baseline cortisol level of 5mcg/dl, among them 70.5% (n=12) had post-synacthen stimulation as inadequate response after sixty minutes while 29% (n=5) showed normal response after sixty minutes. Overall, chi-square test p-value is highly significant (*p*-value <0.001) for this table (Table-III). In addition to this, cross-tabulation of gender and post-synacthen cortisol level was statistically insignificant (p-value = 0.72).

**Table III:** Baseline cortisol levels and post-synacthen cortisol levels.

S. No	Baseline cortisol level mcg/dl	Post-synacthen cortisol after 60 min <20 mcg/dl no. (%)	Post-synacthen cortisol after 60 min ≥20 mcg/dl no. (%)
1.	0 to <1	19 (100)	0 (0.00)
2.	1 to < 2	44 (97.78)	1 (2.22)
3.	2 to <3	20 (83.33)	4 (16.67)
4.	3 to <4	22 (81.48)	5 (18.52)
5.	4 to <5	23 (71.88)	9 (28.13)
6.	5	12 (70.59)	5 (29.41)

## DISCUSSION

In this study we have demonstrated that a morning cortisol value which can be used reliably as an alternative to SST for the diagnosis of PAI is ≤1 mcg/dl (100% sensitivity). As morning cortisol values increases from ≥1mcg/dl to ≤5 mcg/dl it reduces the sensitivity of basal cortisol compared to SST from 98% to 71% respectively. Our study did not show any association of gender with post SST tests. Age difference between groups was also not statistically significant. Our data slightly differs from Endocrine Society Practice guidelines for Adrenal Insufficiency where a morning cortisol value of 3mcg/dl is a reliable cut off for diagnosing PAI^2^.

There are studies favoring strong correlation between morning cortisol and response to Synacthen administration but there is a lack of local data which could be applied on our population.^8^

The SST at a conventional dose of 250 mcg has been validated against the “gold standard” insulin tolerance test (ITT) for diagnosing suspected AI but there are significant challenges to the use of SST in clinical practice including availability of nursing staff, time to perform this test, financial constraints and shortage of synacthen in Pakistan therefore the utility of an alternative test to this dynamic testing cannot be denied.^9^

In western countries where tuberculosis is not endemic the autoimmune Adrenalitis remains the leading cause of PAI and therefore requires a validated assay of auto-antibodies against 21-hydroxylase followed by other tests if the antibody screening is negative thus excluding other causes.^2^ In Pakistan Tuberculosis is still considered as one of the most common causes of PAI.^10^

The need for introducing dynamic testing for diagnosing PAI has been due to the broad range of morning cortisol value i.e. 5–25ug/dL (139–695nmol/L) as the serum cortisol can be affected by stress, exercise and food intake^3^, thus with chances of missing the borderline cases of PAI unless a strong clinical suspicion of adrenal insufficiency. The lack of uniformity in these cut-off levels could in part be credited to differences in study populations, variability of dynamic tests, the use of different serum cortisol assays and dissimilar cut-off peak serum cortisol response indicative of a normal axis response and the difference in the clinical context in which the studies were done. Therefore, Laboratories have to enforce the need to establish reference values for given populations, both for basal or stimulated hormone levels.^11^

In a meta-analysis of 12 studies (635 subjects), a basal cortisol level less than 5 μg/dl (138 nmol/liter) best predicted PAI, whereas values greater than 13 μg/dl (365 nmol/liter) best predicted normal Hypothalamic Pituitary Axis testing hypothalamic-pituitary adrenal insufficiency.^12^

Woods et al in a study of patients with adrenal suppression showed lower cut off values in patients exposed to steroids. A cortisol value of <1.2mcg/dL (<34 nmol/l) having 100% sensitivity for failing an SST and values >12.6 mcg/dL with 100% specificity for passing SST (HPA axis recovery.) thus concluding that using these cut offs 50% of SSTs were performed unnecessarily on patients on inhaled glucorticoids.^13^

The utility of SST for diagnosing PAI cannot be denied although its role in central adrenal insufficiency is still debatable.^14^ But an alternative and simple method of predicting the adrenal cortisol reserves by a morning cortisol level can avoid unnecessary SST testing thus avoiding cost issues and the non-availability of synacthan in different low socioeconomic countries.

We do understand that this is a small data sample and will require further validation by carrying out similar trials in other tertiary care centers in Pakistan but once a consensus is reached regarding the morning basal cortisol cut off values at which PAI can be diagnosed in our population, we can avoid unnecessary SSTs.

### Author`s Contribution

**IU** conceived and designed study, did literature search, collected and analyzed data.

**TA** drafted initial manuscript and helped in literature search.

**AK** helped in analysis and drafting final manuscript.

**NI** supervised the study.
